# Phenotype and functional changes of Vγ9/Vδ2 T lymphocytes in Behçet's disease and the effect of infliximab on Vγ9/Vδ2 T cell expansion, activation and cytotoxicity

**DOI:** 10.1186/ar3043

**Published:** 2010-06-03

**Authors:** Antonina Accardo-Palumbo, Anna Rita Giardina, Francesco Ciccia, Angelo Ferrante, Alfonso Principato, Rosalia Impastato, Ennio Giardina, Giovanni Triolo

**Affiliations:** 1 Department of Internal Medicine, Division of Rheumatology, University of Palermo, piazza delle Cliniche 2, 90127 Palermo, Italy

## Abstract

**Introduction:**

Infliximab is a chimeric monoclonal antibody against tumor necrosis factor alpha (TNF-α) that has been introduced recently for Behçet's disease (BD) patients who were resistant to standard treatment. The aim of this study was to analyse the functional changes of Vγ9/Vδ2 T lymphocytes in both active and inactive disease and the effect of infliximab on Vγ9/Vδ2 T cell expansion, activation and cytotoxicity.

**Methods:**

We investigated 1) cell expansion, 2) expression of TNFRII receptor, 3) perforin and gamma interferon (IFN) content, 4) release of granzyme A (GrA) and 5) phenotype changes, *in vitro *and *in vivo*, in Vγ9/Vδ2 T lymphocytes by means of fluorescence-activated cell sorter analysis of lymphocyte cultures from patients with active and inactive BD and healthy subjects.

**Results:**

Cell expansion, expression of TNFRII, perforin and gamma IFN content and release of granzyme A were significantly higher in active patients. *In vitro *and *ex vivo *treatment with infliximab resulted in a significant reduction of all parameters together with changes in the phenotype of Vγ9/Vδ2 T cells.

**Conclusions:**

All together these data indicate that infliximab is capable of interfering with Vγ9/Vδ2 T cell function in BD and although cell culture models cannot reliably predict all potential effects of the drug *in vivo*, our results present the possibility that this drug may find use in a range of immunological disorders, characterized by dysregulated cell-mediated immunity.

## Introduction

Behçet's disease (BD) is a multisystemic inflammatory disorder characterized mainly by recurrent oral and genital apthous ulcerations and uveitis. The clinical spectrum of BD is wide, involving skin, blood vessels, joints, nervous system, lungs and intestines. The treatment of BD is based for most patients on the combination of corticosteroids and immunosuppressive agents. Despite the improvement obtained with this strategy, relapses and failure may occur, some patients being also refractory to all treatments. Infliximab a chimeric monoclonal antibody against TNF-α that has been introduced for patients with Crohn's disease, rheumatoid arthritis, psoriasis, juvenile chronic arthritis and more recently for BD patients who were resistant to standard treatment [1,2].

The immunopathogenesis of BD remains unknown, but it is believed to be T cell-mediated [3-5]. Recently, attention has been focused on the role of γδ T cells and we have demonstrated that Vγ9/Vδ2 circulating lymphocytes are activated in patients with active disease and express increased levels of receptors for TNF-α and IL (interleukin)-12 [6]. Moreover, elevated levels of granzyme A both in the serum and Vγ9/Vδ2 cell supernatants of active BD patients are present, suggesting a role for this kind of lymphocytes in the pathogenesis and in the progression of the disease [7]. In this paper we analysed the functional changes of Vγ9/Vδ2 T lymphocytes in both active and inactive disease and of the effect of infliximab on Vγ9/Vδ2 cell expansion, activation and cytotoxicity.

## Materials and methods

### Patients

Thirteen patients with BD (nine males and four females, mean age 42 ± 24 years), classified according to the International Study Group for Behçet's Disease [8] were studied. The activity of BD was assessed by collecting clinical symptoms defined according to the BDCAF score [9,10] that includes the presence of several manifestations of the disease, by the uveitis scoring system and by the visual activity measurement [11]. At the time of sampling, disease was active in six patients and inactive in seven. In five of the active patients blood for serum and lymphocyte studies was obtained before and after the anti-TNF-α (Infliximab) therapy. All patients were using colchicine (n = 13), and/or low dose corticosteroids (n = 8). Ten healthy volunteers (age range 21 to 47, mean 30 ± 8 years) were enrolled as controls. None of patients or controls were HIV, CMV, EBV infected. Human studies committee approval and individual informed consent from each patient were obtained.

### Cell separation and in vitro expansion by Vγ9/Vδ2 T lymphocytes

Peripheral blood mononuclear cells (PBMC) were obtained from each individual by separating heparinized venous blood on Ficoll (Euroclone, Wetherby, Yorkshire, UK). The cells were washed in RPMI-1640 medium (Euroclone), and cultured in 24-well plates (Costar, Cambridge, MA, USA) at a concentration of 5 × 10^5 ^cells/ml in RPMI-1640 supplemented with 10% foetal calf serum (Euroclone) 20 mM Hepes (Euroclone), 2 mM L-glutamine (Euroclone) and 100 U/ml penicillin/streptomycin (Sigma, St Louis, MO, USA) at 37°C at 0,5% CO2. For the expansion of Vγ9/Vδ2 T cells, PBMCs were cultured for 10 days in medium alone or in the presence of 0,5 mM Dimethylallyl pyrophosphate (DMAPP, Sigma, St Louis). After 72 hours, cultures were supplemented with a 0,5 ml medium containing 40 U/ml recombinant human IL-2 (Genzyme, Cambridge, MA, USA). Every 72 h, 0.5 ml medium was replaced with a 0.5 ml fresh medium containing IL-2. After 10 days, cells were washed three times in medium, and expansion of Vγ9/Vδ2 T cells was assessed using a FACScan flow cytometer (Becton Dickinson, Mountain View, CA, USA) by using forward scatter/side scatter gating to select the lymphocyte population for analysis. The Vγ9/Vδ2 T cell expansion factor (EF) was then calculated as described above [6].

### Monoclonal antibody and flow cytometry

Monoclonal antibodies (MOAbs) specific for human surface antigens anti-T cell receptor (TCR) Vδ2 fluorescein isothiocyanate-labelled (FITC; PharMigen, San Diego, CA, USA), anti-CD27 phycoerythrin-labelled (PE, PharMigen), anti-CD45RO quantum red (QR, Sigma), anti-TNF-RII PE (R&D System, Minneapolis, MN, USA) were used. For the evaluation of intracytoplasmic content of perforin (Pf) and IFN-γ, 3 × 105 cells were stained with anti- Vδ2 TCR FITC and, after washing, fixed with 4% paraformaldehyde (Sigma) for 30 minutes at 4°C. After two washes with permeabilization buffer (saponine containing) the cells were incubated at 4°C for 45 minutes with anti perforin Pe antibody (Ancell Corporation, Bayport, MN, USA) or with anti-IFN-γ PE (Euroclone). After washing, the cells were suspended in PBS with 1% foetal calf serum and data were acquired on a FACScan instrument and analyzed using WinMDI version 2.8 software.

The number of Pf and TNF-RII molecules (MESF; molecular equivalents of soluble fluorochrome) was calculated by fluorescence-activated cell sorter analysis of cells stained with saturating amounts of PE labelled anti-TNF-RII and anti-Pf mAb of known PE/protein ratio and comparing the staining with a standard curve of microbeads labelled with defined numbers of PE molecules (Quantum Fluorescence Kit, Sigma). The analysis was done using Quickal Program for MESF Units for Windows.

### Esterase assay for GrA

GrA activity was tested using the synthetic substrate N-α-benzyloxycarbonyl-L-lysine thiobenzyl ester (z-lys-SBzl; Sigma) as previously described [7]. Briefly, 20 μl of supernatants (obtained at the 10^th ^day of Vγ9/Vδ2 culture) or diluited (1:100 in PBS) sera were coincubated with 35 μl 1 mM z-lys-SBzl and 35 μl 1 mM 5,5-dithio-bis-(2nitrobenzoic acid) (Sigma). After incubation at 37°C for 2 hours and 30 minutes respectively, the absorbance at 405 nm was determined. Esterolytic activity of GrA was reported as mOD units.

### In vitro effect of Infliximab on Vγ9/Vδ2 cultures

In order to examine the effects on Vγ9/Vδ2 expansion, TNF-RII expression, perforin and IFN-γ content, and supernatant levels of GrA, Infliximab (Remicade; Centocor Inc., Malvern, PA, USA; Schering Plough SpA, Segrate (Mi), Italy) was added in the medium at a final concentration of 10, 50 (for 3 days) and 100 μg/ml (for 3 and 10 days).

### Effect of Infliximab therapy

Five patients with active disease were treated with Infliximab, 5 mg/Kg, by a two hour infusion, at weeks 0, 2, 4 and the patients observed for a further two hours without adverse effects. Sampling, for Vγ9/Vδ2 studies, were performed before the start of therapy and at the time of the second infusion.

### Statistics

All values are expressed as mean ± SD. We performed analysis of significance in Prism (GraphPad, La Jolla, CA, USA) by the two-tailed t test analysis and by two-way ANOVA.

## Results

### Functional changes of Vγ9/Vδ2 T lymphocytes

Cumulative results are shown in Table 1. The EF of Vγ9/Vδ2 T lymphocytes from active BD patients was significantly higher (295 ± 44; *P *<0.0001) than those obtained from inactive patients (36 ± 8) and from healthy controls (30 ± 8). Vγ9/Vδ2 TNF-RII was 10,422 ± 1,694 in active patients, 4,087 ± 1,671 in inactive patients and 4,512 ± 1,436 in healthy controls. The intracytoplasmic content of IFN-γ was 40.4 ± 8.2% in Vγ9/Vδ2 standard cultures from active patients, and 18 ± 7% and 11 ± 4% in inactive patients and healthy subjects respectively. Intracytoplasmic perforin content was 27,581 ± 4,611 in active patients and, 5,247 ± 1,230 and 4,980 ± 1,110 in inactive patients and controls respectively.

**Table 1:** Functional changes of Vγ9/Vδ 2 T lymphocytes in BD patients and controls

	Active BD	Inactive BD	Controls	*P*
EF of Vγ9/Vδ2	295 ± 44	36 ± 8	30 ± 8	< 0.0001
Vγ9/Vδ 2 TNF-RII	10,422 ± 1,694	4,087 ± 1.671	4,512 ± 1436	< 0.0001
IFN-γ (% of Vγ9/Vδ 2)	40.4 ± 8.2	18 ± 7	11 ± 4	< 0.001
Perforin (mOD units)	27,581 ± 4.611	5,247 ± 1,230	4,980 ± 1,110	< 0.001
Granzyme A (mOD units)	818 ± 97	589 ± 21	550 ± 30	< 0.05

BD: Behçet's disease; EF: Expansion factor.

GrA levels were (818 ± 97 mOD units) significantly higher in the supernatants obtained from cultures of active patients than those obtained from inactive patients (589 ± 21) and controls (550 ± 30). Staining of PBMC cultures with antibodies to Vδ2-TCR, CD45RO and CD27 identified four subsets of Vγ9/Vδ2 T cells (Table 2): 1) a subset with a naive CD45RO^- ^CD27^+ ^phenotype, 2) a subset with a memory CD45RO^+ ^CD27^+ ^phenotype; 3) a subset with a effector CD45RO^+ ^CD27^- ^phenotype and 4) a subset with a terminally differentiated cytotoxic CD45RO^- ^CD27^- ^phenotype. In active patients we observed that the phenotype of Vγ9/Vδ2 T cells is mainly composed of CD45RO^+^CD27^- ^effector cells (57 ± 13%) and that there is, also, evidence of CD45RO^-^CD27^- ^terminally differentiated cytotoxic cells (20 ± 6%). In contrast, in Vγ9/Vδ2 cultures of inactive patients as well as of normal controls, we found mainly the phenotypes CD45RO^+^CD27^+ ^(47 ± 9 and 49 ± 9%) and CD45RO^- ^CD27^+ ^(24 ± 7 and 20 ± 8%) that are characteristics of memory and naive cells, respectively.

**Table 2:** Subpopulations of Vγ9/Vδ 2 T lymphocytes in BD patients and controls

	Active BD	Inactive BD	Controls	*P*
CD45RO^-^CD27^+ ^(%)	7.5 ± 2.25	24 ± 7	28 ± 8	< 0.0001
CD45RO^+^CD27^+ ^(%)	11 ± 6.44	47 ± 9	49 ± 9	< 0.0001
CD45RO^+^CD27^- ^(%)	57 ± 13	17.8 ± 9.7	19.2 ± 6.7	< 0.001
CD45RO^-^CD27^- ^(%)	20 ± 6	8.6 ± 3.8	5.5 ± 5.5	< 0.001

BD, Behçet's disease.

### In vitro studies with Infliximab

**Figure 1:**
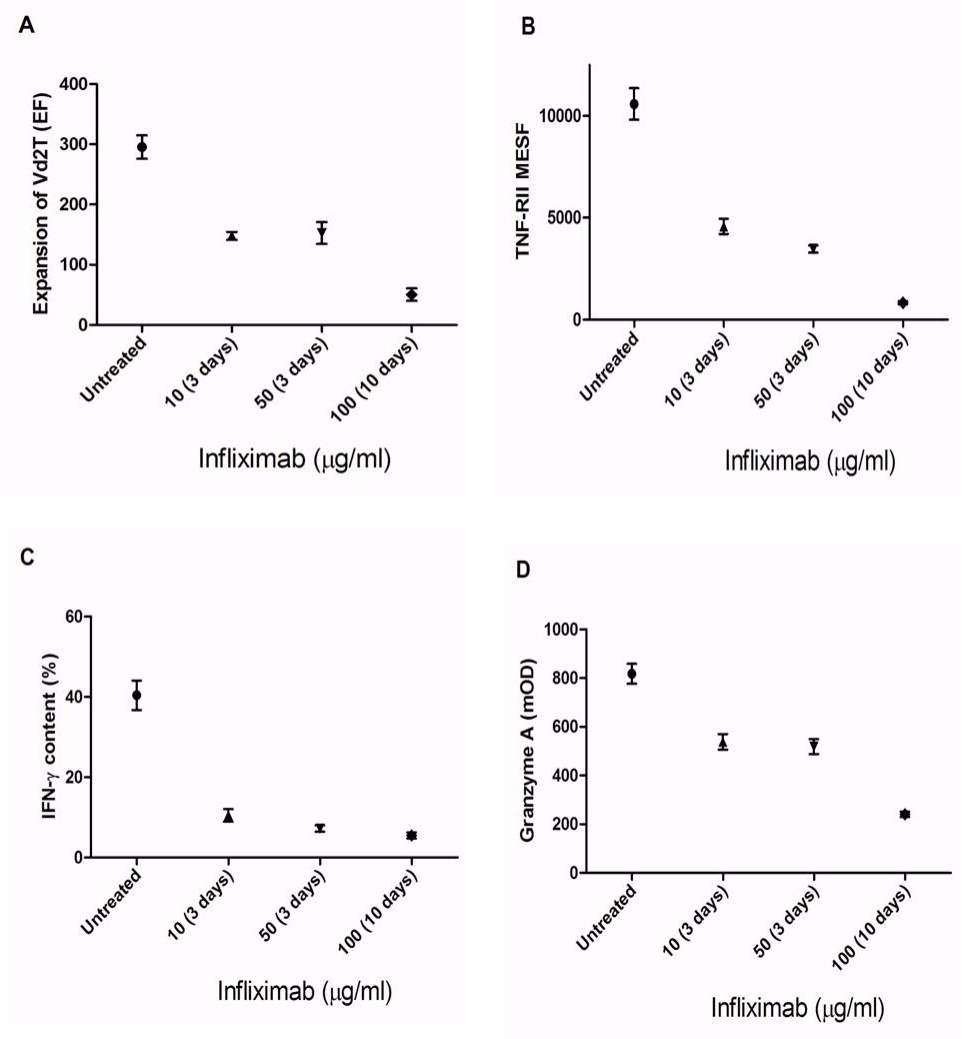
**Effects of *in vitro *addition of Infliximab (10, 50 and 100 μg/ml) to the cultures from active patients**. **(a) **Expansion of Vγ9Vδ2 T lymphocytes (%); **(b) **TNF-RII (MESF); **(c) **IFN-γ content (%) and **(d) **granzyme release (mOD).

The addition of Infliximab to the cultures from active patients determined a significant time and dose dependent inhibition of cell expansion (Figure 1a) and a significant decrease of TNF-RII expression (Figure 1b). In particular, the mean EF was 295 ± 43.4 in untreated cultures and 50 ± 23 in culture with the highest infliximab concentration (*P *< 0.001). TNFRII MESF was 10,500 ± 1,707 in untreated culture and 4,576 ± 845 (Infliximab 10 μg/ml for 3 days; *P *< 0.05), 3,466 ± 394 (Infliximab 50 μg/ml for 3 days; *P *< 0.01) and 833 ± 165 (Infliximab 100 μg/ml for 10 days, *P *< 0.001) respectively after exposure to infliximab. IFN-γ in untreated cultures was 40.4 ± 8.25%. In the presence of Infliximab we found values of 10.5 ± 3.51% (Infliximab 10 μg/ml for 3 days; *P *< 0.01), 7.2 ± 1.8% (Infliximab 50 μg/ml for 3 days; *P *< 0.001) and 5.5 ± 1.7% (Infliximab 100 μg/ml for 10 days; *P *< 0.001) (Figure 1c).

**Figure 2:**
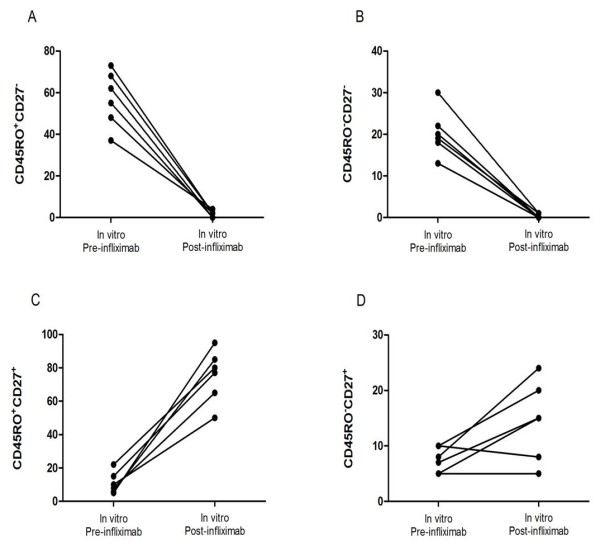
**Percentage of Vγ9/Vδ2 cells isolated from active patients cultured in presence of Infliximab (50 μg/ml)**. **(a) **Effector CD45RO^+^CD27^-^; **(b) **Cytotoxic CD45RO^-^CD27^-^; **(c) **Memory CD45RO^+^CD27^+^; naive CD45RO^-^CD27^+^.

A significant reduction of cell perforin content (7,040 ± 985 vs 27,573 ± 4,590, *P *< 0.001) was also observed after infliximab (50 μg/ml for 10 days) (not shown) exposure together with a decrease of granzyme release in the supernatants (Figure 1d); in standard cultures the GrA levels were 818 ± 91.6 mOD, in the presence of Infliximab the levels were 538 ± 72 (10 μg/ml for 3 days; *P *< 0.05), 518 ± 69 mOD (50 μg/ml for 3 days; *P *< 0.05) and 240 ± 25 mOD (100 μg/ml for 10 days; *P *< 0.01). The Vγ9/Vδ2 cells from active patients cultured in presence of Infliximab (50 μg/ml) shown mainly the phenotype of memory (75 ± 15%) and naive (21 ± 3%) cells rather than those of effector (1.76 ± 1.28%) and cytotoxic (0.3 ± 0.21%) cells (Figure 2).

### In vivo treatment with Infliximab

Five patients with active disease were infused with 5 mg/Kg of Infliximab, and Vγ9/Vδ2 studies were performed before and after therapy (Table 3). A significant EF difference was found in the Vγ9/Vδ2 cultures from patients before (256 ± 90) and after (80 ± 44; *P *< 0.01) Infliximab therapy. Serum GrA levels were 715 ± 174 mOD and 207 ± 72 mOD (*P *< 0.001) respectively. After therapy, the cells showed the phenotype of memory (61 ± 11%) and naive (21 ± 3%) cells.

**Table 3:** Effects of in vivo administration of infliximab on BD patients with active disease

	BD pre-infliximab	BD post-infliximab	*P*
Serum Granzyme mOD	715 ± 74	207 ± 72	< 0.001
EF of Vγ9/Vδ2	256 ± 90	80 ± 44	< 0.001
CD45RO^-^CD27^+ ^(%)	4.4 ± 3.6	21 ± 2.6	< 0.01
CD45RO^+^CD27^+ ^(%)	9.2 ± 7.5	61.4 ± 11.1	< 0.001
CD45RO^+^CD27^- ^(%)	54 ± 12	7 ± 3	< 0.0001
CD45RO^-^CD27^- ^(%)	20.6 ± 22	6 ± 6.5	< 0.001

BD, Behçet's disease.

## Discussion

The immunopathogenesis of BD is unknown. Various micro-organisms such as streptococci and herpes simplex virus have been implicated in the pathogenesis in genetically susceptible HLA-B51+ individuals [12,13].

There is evidence of immunological dysregulation, including neutrophil hyperfunction [14,15], autoimmune manifestations [16] and several phenotypic and functional lymphocyte abnormalities, most of the immunological studies suggesting a central role for T cells in the pathogenesis of this disease [17]. Cytotoxic T cells are considered to play a role in the development of disease. Recent studies [18,19] and our own [6] point also for a role of activated Vγ9/Vδ2 T lymphocytes in the progression and probably in the pathogenesis of the disease.

The treatment of BD comprises mainly systemic corticosteroids for most manifestations of BD. Supplemental therapy with other immunomodulatory agents is often necessary to control serious manifestations such as uveitis and meningoencephalitis and to reduce the incidence of long-term steroid toxicity. Drugs usually used in BD such as glucocorticoids, pentoxifylline and cyclosporine have been demonstrated to modulate peripheral blood gamma delta T lymphocytes [20-22].

The role of TNF-blocking agents on gamma delta T lymphocyte functions has not yet been investigated. In BD, increased serum levels of TNF-α and soluble TNF-RII has been observed during the active stage of disease suggesting a role for TNF-α in the pathogenesis [23-25]. In addition, TNF-α, that has been reported also to be produced by γδ T cells, might stimulate the TNF receptor bearing γδ T cells, in an autocrine or paracrine manner or both, to proliferate [26,27].

In this study we investigated the functional changes of Vγ9/Vδ2 T lymphocytes in both active and inactive Behçet's disease and the effect of Infliximab on Vγ9/Vδ2 T cell expansion, activation and cytotoxicity.

Infliximab is a high affinity monoclonal anti TNF-α antibody that has been introduced for Crohn's disease and rheumatoid arthritis treatment in patients who are resistant to standard therapy. Our previous studies demonstrated a complete remission of all disease manifestations in BD patients with ocular involvement and cerebral vasculitis [1,2].

Infliximab interferes with Vγ9/Vδ2 T cell functions. In particular, *in vitro *and *in vivo *studies demonstrated that this drug was able to suppress the Vγ9/Vδ2 T cell expansion and activation (TNF-RII expression and IFN-γ production) induced by DMAPP. Infliximab interferes also with the potential cytotoxic activity of these cells that we evaluated through the expression of the cytoplasmic granule-associated molecules perforin and the GrA release in the medium of cultures.

In this paper we did not study the phenotype of circulating Vγ9/Vδ2 T lymphocytes being our cytofluorimetric analysis not sensitive enough to measure membrane antigens in a relatively low number of cells. Although these preliminary observations have to be properly defined, after phosphoantigen stimulation, however, we observed frequently that the Vγ9/Vδ2 subpopulation was mainly composed by effector cells in active patients and by memory cells in inactive patients and controls. After *in vitro *exposure to Infliximab, there was a lack of effector cells, suggesting that this drug might block the lineage pattern of differentiation (naive → memory → effector → terminally differentiated cells), as a consequence of expansion and activation inhibition.

## Conclusions

Our observations of disease-specific changes in Vγ9/Vδ2 T cell functions are consistent with the hypothesis that these cells play a role in the pathogenesis of BD. Despite the relatively low number of patients enrolled, data collected in this study point also to a critical role in the regulation of cellular activation and function of Vγ9/Vδ2 T cells by Infliximab and lead to encourage the possibility that this drug may find widespread use in the treatment of BD.

## Abbreviations

BD: Behçet's disease; EF: expansion factor; FITC: fluorescein isothiocyanate; GrA: granzyme A; MESF: molecular equivalents of soluble fluorochrome; MoAbs: monoclonal antibodies; PBMCs: peripheral blood mononuclear cells; PE: phycoerythrin; Pf: perforin; z-lys-sBzl: N-a-benzyloxycarbonyl-L-lysine thiobenzil ester; TCR: T cell receptor; TNF-α: tumor necrosis factor alpha.

## Competing interests

The authors declare that they have no competing interests.

## Authors' contributions

AA-P participated in the study design, acquisition of data, analysis and interpretation of data, manuscript preparation. ARG participated in the study design and analysis and interpretation of data, manuscript preparation. FC participated in the study design, acquisition of data, analysis and interpretation of data, manuscript preparation and carried out statistical analysis. AF, AP, RI and EG participated in the study design, and analysis and interpretation of data. GT participated in the study design, acquisition of data, analysis and interpretation of data, manuscript preparation and carried out overall study supervision.
